# Improving Anti-Corrosion Property of Aluminium Alloy by Fabrication MAO Coating via Mixing Titanium Potassium Oxalate into Electrolyte

**DOI:** 10.3390/molecules30010153

**Published:** 2025-01-02

**Authors:** Wei Song, Yasheng Xing, Zhen Song

**Affiliations:** 1 School of Biological and Chemical Engineering, Nanyang Institute of Technology, No. 80 Changjiang Road, Nanyang 473004, China; song78wei@163.com; 2 Henan Key Laboratory of Industrial Microbial Resources and Fermentation Technology, Nanyang Institute of Technology, Nanyang 473004, China; 3 Nanyang Branch of Henan Boiler and Pressure Vessel Inspection Technology Research Institute, No. 1088 Gongye South Road, Nanyang 473000, China

**Keywords:** aluminium alloy, anti-corrosion, titanium potassium oxalate, micro arc oxidation, coating

## Abstract

Titanium potassium oxalate had been mixed into the electrolyte to improve the anti-corrosion property of the micro arc oxidation coating on the surface of the aluminium alloy. The surface and cross-section of the coating at different titanium potassium oxalate concentrations had been observed by scanning electron microscopy, showing that when the titanium potassium oxalate concentration was 10 g/L, the coating compactness was better. Additionally, the element content of the coating had been studied by the energy dispersive spectrometer, and results proved that the coating consisted of Al, O, Ti, Si, and P. The Ti increased with the increase in titanium potassium oxalate concentration. The X-ray diffractometer had been employed to analyze the crystalline structure of the coating. Then, it was found that after the micro arc oxidation, an alumina oxide coating was prepared on the surface of the aluminium alloy, and the Al_2_O_3_ and TiO_2_ characteristic peak was observed. Furthermore, the electrochemical workstation was used to test the anti-corrosion of the coating. It was proved that when the titanium potassium oxalate concentration was 10 g/L, the open circuit voltage, corrosion current, corrosion potential, and impedance of the coating were improved, and the anti-corrosion property of the aluminium alloy had been strengthened.

## 1. Introduction

To protect the environment and save energy, electric vehicles have become the trend of automobile development. However, the insufficient mileage of electric vehicles restricts their application. The battery capacity cannot increase indefinitely because the mileage is the ratio of battery capacity to energy consumption and is affected by driving safety and body size [1]. The greater the power consumption per 100 km, the shorter the battery consumption time, and the shorter the mileage of electric vehicles. Considering that a heavier car means a higher energy consumption, an effective method of increasing the mileage is to lower the weight of the vehicle.

The battery weight of electric vehicles is about 28% of the total vehicle weight, and to ensure the stable operation of electrical appliances, copper is mostly used as the motor component. If light metal has been used to replace copper motor components, the quality of the vehicle can be improved, and the mileage can be increased dramatically. Aluminium alloy is characterized by high specific strength, strong thermal conductivity, and good decorative performance, thus being widely used in electronics and electricians, transportation, aerospace, construction, chemical industry, and many other fields [2,3]. The anti-corrosion of the materials is an important factor to consider in their application. Many factors, such as environmental and material characteristics, and the morphology and structure of metal surfaces, affect their anti-corrosion. As an important component in electric vehicles, the external environment of aluminium alloy application is uncontrollable. In order to ensure the stable quality of the device and reduce costs, commercial products such as the 6××× series aluminium alloy should be used to prepare components. Therefore, improving the morphology and structure of aluminium alloy surfaces has become a reasonable way to enhance their anti-corrosive properties. However, aluminium is an active metal, and its corrosion potential is only −1.66 V, which is far lower than that of copper. However, an aluminium coating can be formed on the surface of the aluminium alloy to protect the substrate from further corrosion. Because the thickness of the coating is only 2 μm, the alloy compositions in the aluminium alloy affect the integrity of the coating. It is difficult to achieve excellent corrosion resistance for the naturally formed aluminium oxide coating on the surface of the aluminium alloy. The insufficient corrosion resistance impedes the wide application of aluminium alloy in harsh environments [4]. Valve metals are conductors, while their oxides are insulators. Using aluminium, magnesium, titanium, and other valve metals or their alloys as the anode and placing them in the electrolyte, a very thin matrix oxide insulating coating is immediately formed on the surface of the matrix after energization. When the voltage exceeds a certain value, the lowest resistance value in the coating is broken down, and the oxygen plasma in the electrolyte generates an in situ micro arc discharge phenomenon. Under the strong exothermic effect of in situ oxygen plasma, the crystalline oxide coating (MAO coating) can be prepared and grown on the surface of the valve metal or their alloy. With the breakdown of the insulation coating, the elements in the electrolyte penetrate into the coating, and finally become a component of the oxide coating. When the micro arc discharging time is very short (about 0.1 ms), and because the breakdown point is always transferred in the lowest resistant position, the MAO coating on the surface of the valve metal is uniform. The reaction of the MAO coating formation is shown as Formulas (1)–(4):

Cathode:

2H^+^ = H_2_ (1)

Anode:

Al^3+^ + 3 OH^−^ = Al(OH)_3_     ∆H = −77.1 KJ/mol (2)

2 Al(OH)_3_ = Al_2_O_3_ + 3 H_2_O     ∆H = +194.6 KJ/mol (3)

4 OH^−^ + 4 e^−^ = 2 H_2_O + O_2_     ∆H = + 46.6 KJ/mol (4)

Compared with Al, Al_2_O_3_ has better corrosion resistance if a compact coating can be prepared on the surface of Al, and its corrosion resistance could be improved [5]. Nevertheless, as the crystalline coating is grown under the action of the outer surface of the valve metal, the breakdown temperature of the micro arc is as high as 10^3^–10^4^ K. Under the action of the high temperature, the MAO coating is full of micro pores/cracks, reducing the corrosion resistance of the coating.

It had been widely reported that doping nano TiO_2_ in the electrolyte can regulate the crystalline composition of the MAO coating, reduce the size and number of the micro pores/cracks in the coating, and then improve the corrosion resistance of the coating [6,7]. Because nano TiO_2_ is insoluble in the electrolyte, the doping amount of TiO_2_ is low, and the improvement of coating corrosion resistance is limited. The typical electrolyte system for preparing MAO coating on the surface of aluminium alloy is composed of the aqueous solution of sodium silicate, oxalic acid, potassium hydroxide, and other components [8,9,10]. If potassium titanium oxalate is dissolved in the electrolyte, and potassium titanium oxalate hydrolysis is facilitated under the action of the electric field force, forming nano titanium dioxide sol and participating in the coating formation, the doping amount of nano titanium dioxide in the MAO coating can be improved, and the corrosion resistance of the coating can be enhanced. Based on the above analysis, to expand the application fields of aluminium alloys, its anti-corrosion property must be improved. Ti ions can effectively decrease the micro cracks and pore sizes of MAO coatings, which is beneficial to improve the anti-corrosion of MAO coatings. To increase the Ti icon content in the MAO coating, potassium titanium oxalate had been mixed into the electrolyte, and SEM, XRD, and EDS analyses had been carried out to investigate the composition of MAO coatings under different potassium titanium oxalate doping conditions. The open circuit potential, corrosion current, corrosion potential, and impedance were analyzed by the electrochemical workstation to determine the appropriate doping amount of potassium oxalate, aiming to improve the corrosion resistance of MAO coatings on the surface of the aluminium alloy.

## 2. Results and Discussion

### 2.1. Influence of Titanium Oxalate Potassium Concentration on the Micro Surface Morphology of the Coating

**Figure 1:**
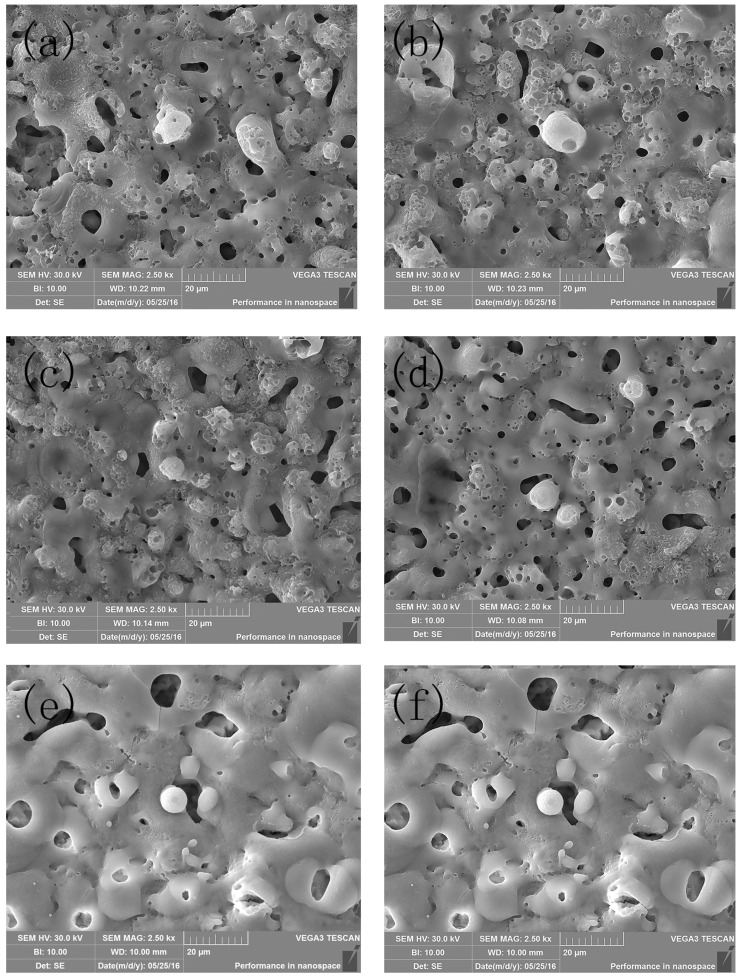
The SEM photos of the surface coatings at titanium potassium oxalate concentration of (**a**) 0 g/L, (**b**) 5 g/L, (**c**) 8 g/L, (**d**) 10 g/L, (**e**) 12 g/L, and (**f**) 15 g/L.

Figure 1 illustrates the SEM photos of the surface coatings. It can be seen from Figure 1a that without mixing the titanium oxalate potassium into the electrolyte, the surface of the micro arc oxide coating was rough and uneven. The distribution of the pore size in the micro arc oxide coating was uneven. When the titanium oxalate potassium was mixed into the electrolyte (see Figure 1b–f), the distribution of the pore size in the coating became uniform, and the pore quantity in the coating decreased.

**Figure 2:**
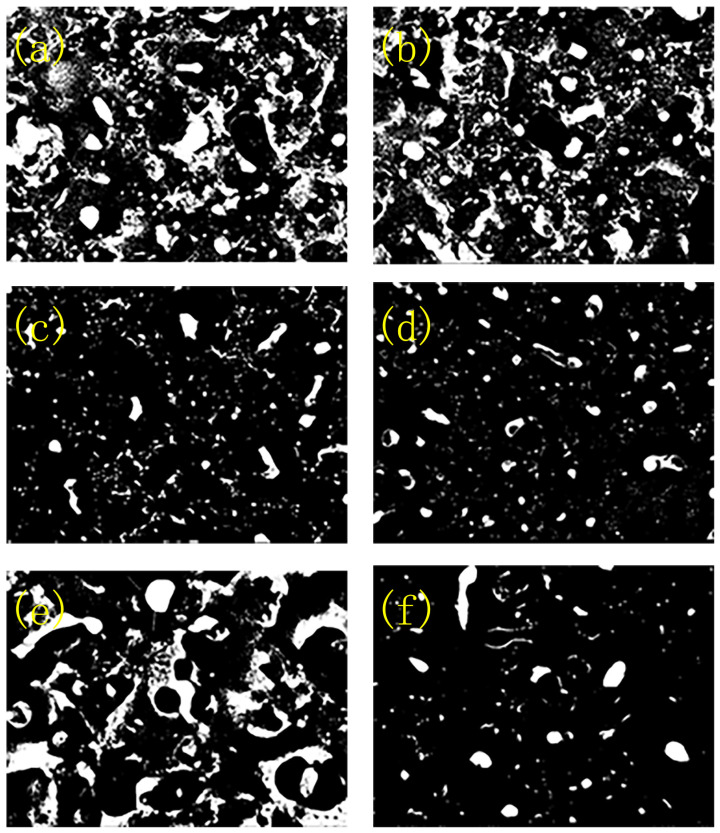
The Image J photos of the surface coatings at titanium potassium oxalate concentration of (**a**) 0 g/L, (**b**) 5 g/L, (**c**) 8 g/L, (**d**) 10 g/L, (**e**) 12 g/L, and (**f**) 15 g/L.

To further investigate the micro crack and micro pore on the surface of the sample, Image J software (Version 1.51) had been used to study the pore and crack. Results are shown in Figure 2. It can be seen that the pore and crack on the surface decrease with the increase in titanium oxalate potassium dosage. Specifically, when the titanium oxalate potassium concentration reached 10 g/L, the surface coating was more compact and uniform, mainly because the [TiO(C_2_O_4_)_2_]^2−^ in the electrolyte decreased the arc starting voltage, and it could form a complexation with the other icon, which could decrease the arc size and increase the arc quantity. As a result, a compact and uniform coating could be prepared on the surface of the substrate.

**Figure 3:**
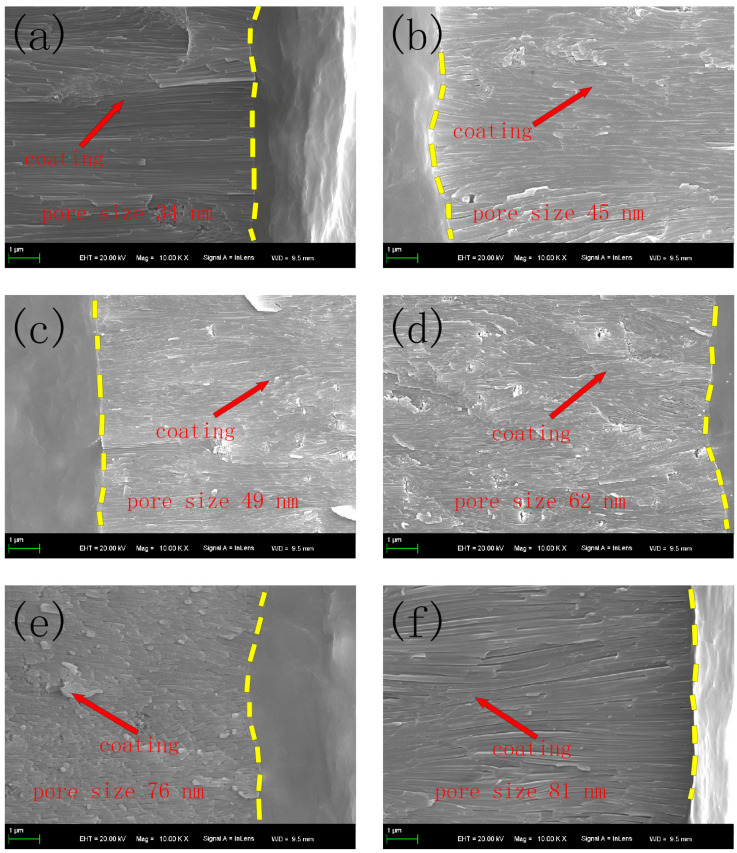
The cross-section of the coatings at titanium potassium oxalate concentrations of (**a**) 0 g/L, (**b**) 5 g/L, (**c**) 8 g/L, (**d**) 10 g/L, (**e**) 12 g/L, and (**f**) 15 g/L.

Figure 3 shows the cross-section photos of the coating. It can be observed that with the increase in titanium oxalate potassium concentration, the pore size of the coating increased. Because the ceramic coating was formed on the action of micro arc discharging [11], when the pore size increased, the discharge channel was smooth, and the crystalline alumina was easily formed. However, when the titanium oxalate potassium concentration reached 15 g/L, the discharge channel was too big to decrease the compactness of the coating. In general, the greater the compactness of the coating, the higher its anti-corrosion. Therefore, the suitable titanium oxalate potassium concentration of 10 g/L was selected.

### 2.2. Influence of Titanium Oxalate Potassium Concentration on the Crystalline of the Coating

**Figure 4:**
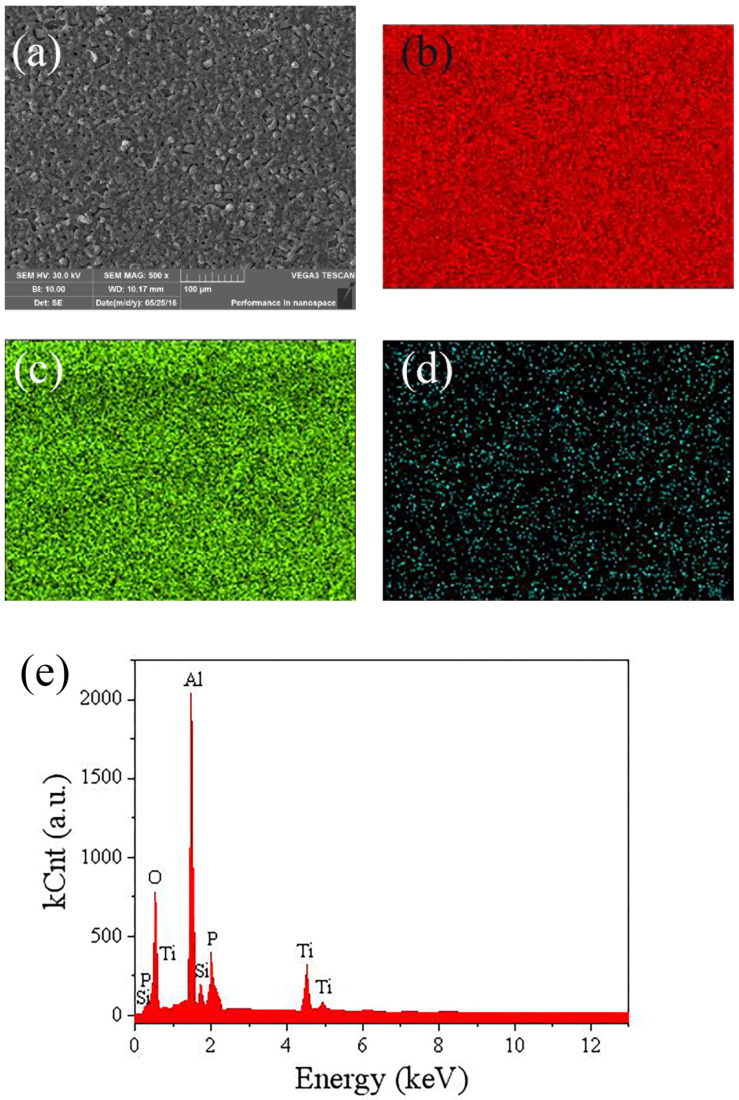
(**a**) The micro surface morphology of the coating, the element distribution of (**b**) Al, (**c**) O, and (**d**) Ti, and the (**e**) element content in the coating.

Figure 4 displayed the EDS spectrum of the coating. Figure 3a illustrated the micro surface photos of the coating, while Figure 3b–d showed the distribution of Al, O, and Ti elements in the coating. It can be observed that Al, O, and Ti elements were uniformly distributed throughout the coating. In Figure 3e, the element content in the coating is shown. To be specific, it can be seen that there were Al, O, Si, and Ti elements in the coating, while the Al content was significantly higher than the other element. In addition, there were P and Si elements in the coating.

The element content of the coating at different titanium oxalate potassium concentrations is presented in Table 1. It can be found that there were Al, O, Ti, Si, and P elements in the coating, proving the coatings were composed of Al_2_O_3_, TiO_2_, P_3_O_4_, and SiO_2_. As the Ti, P, and Si elements were the components of the electrolyte, it was demonstrated that the electrolyte component was involved in the composition of the micro arc oxidation coating [12]. With the increasing titanium oxalate potassium concentrations, the Al, O, and Si contents decreased slightly, while the Ti content increased in the coating.

**Figure 5:**
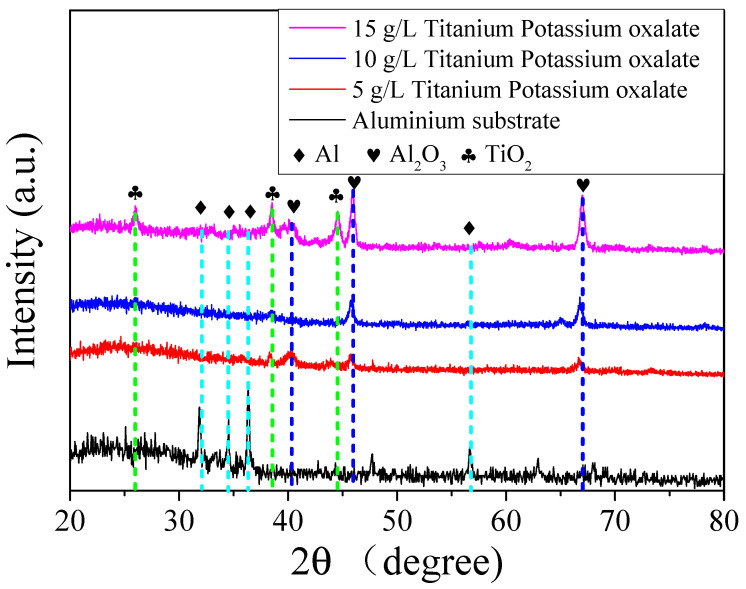
The XRD spectrum of the coatings.

The X-ray diffractometer spectrum of the Al substrate and Al_2_O_3_ coating prepared at the titanium oxalate potassium concentrations of 10 g/L and 15 g/L are indicated in Figure 5. It can be found that at 32.5°, 34.8°, 36.3°, and 56.7°, there were Al characteristic peaks appearing when the sample was the Al substrate [13]. When the micro arc oxidation coating was prepared, at 40.2°, 44.8°, and 67.2°, there were Al_2_O_3_ characteristic peaks [14]. At 26.2°, 38.4°, and 44.1°, TiO_2_ characteristic peaks appeared [15]. Thus, it was proved that after micro arc oxidation, an alumina oxide coating could be prepared on the surface of the Al substrate. When the titanium oxalate potassium was added into the electrolyte, there was TiO_2_ content in the coating. Furthermore, the peak strength of Al_2_O_3_ and TiO_2_ increased with the increase in titanium oxalate potassium concentration.

### 2.3. Influence of Titanium Oxalate Potassium Concentration on the Open Circuit Voltage of the Coating

**Figure 6:**
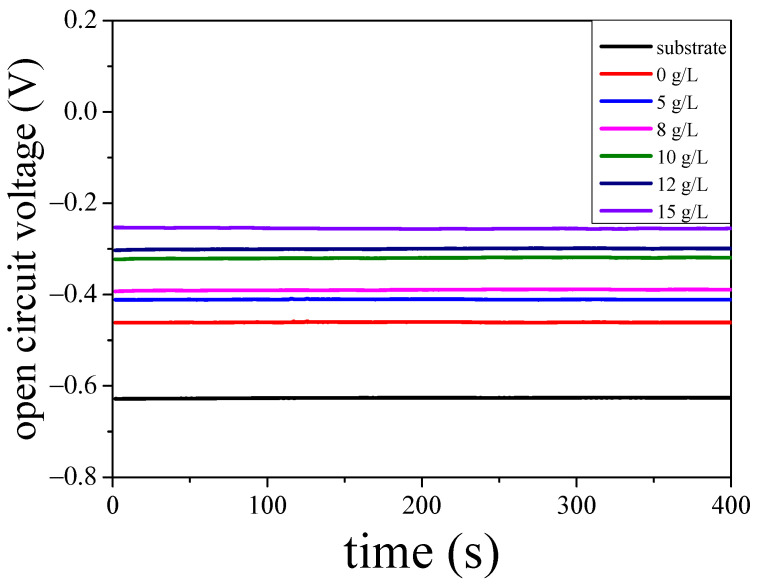
The open circuit voltage of the coatings.

The open circuit voltage of the aluminium alloy substrate, and the micro arc oxidation coating at titanium potassium oxalate concentrations of 0 g/L, 5 g/L, 8 g/L, 10 g/L, 12 g/L, and 15 g/L were investigated using the electrochemical workstation. The results are demonstrated in Figure 6. It can be observed that after the micro arc oxidation coatings were prepared on the surface of the substrate, its open circuit voltage increased significantly. The open circuit voltage increased with the increase in titanium potassium oxalate concentration, because the anti-corrosion of the samples is positively correlated with its open circuit voltage [16]. The results proved that the anti-corrosion of the aluminium alloy can be strengthened by using the micro arc oxidation method; in addition, by mixing titanium potassium oxalate into the electrolyte, the anti-corrosion of the micro arc oxidation coating could be further improved.

### 2.4. Influence of Titanium Oxalate Potassium Concentration on the Corrosion Current and Corrosion Potential of the Coating

**Figure 7:**
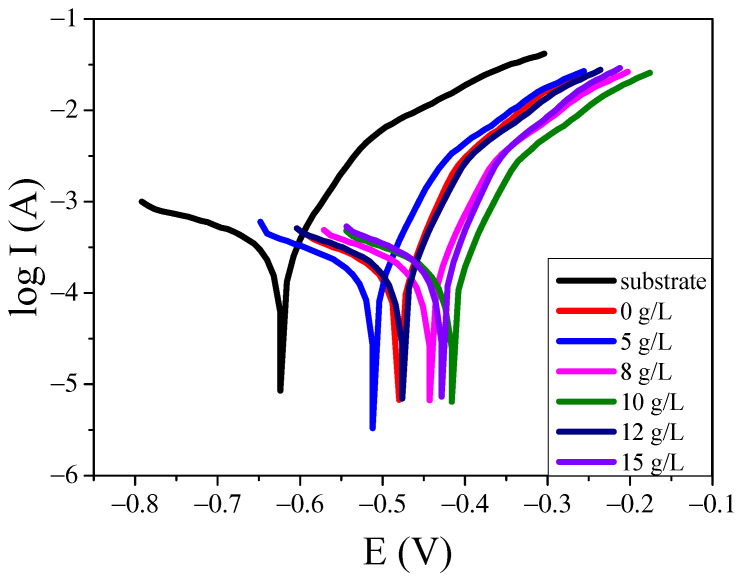
The corrosion current and corrosion potential of the coatings.

The anti-corrosion of the coating had been studied using the electrochemistry station, and the results are shown in Figure 7. It can be seen that before the samples were treated by MAO technology, the corrosion current was large. After the MAO coatings were prepared, the corrosion current decreased significantly. It is well known that the anti-corrosion performance of the aluminium alloy increases with the decrease in its corrosion current [17]. Therefore, the anti-corrosion of the aluminium alloy can be enhanced using MAO technology. The corrosion current of the coatings decreases with the increase in potassium titanium oxalate concentration, demonstrating that the TiO_2_ in the coatings improved the anti-corrosion property of the coating. However, when the potassium titanium oxalate concentration reached 15 g/L, the polarization current of the coating decreased slightly. The results indicated that the potassium titanium oxalate in the electrolyte improved the compactness of the coating. The probable reason is that at the action of the electric field force, the complex ion [TiO(C_2_O_4_)_2_]^2−^ can be migrated to the surface of the anode. Finally, a complex ion enrichment area around the anode area was formed, thus reducing the discharge arc length and enhancing the compactness of the coating.

The corrosion potential and corrosion current of the coatings at different potassium titanium oxalate concentrations were tested and are indicated in Table 2. It can be seen that when the potassium titanium oxalate concentration was 0 g/L, the corrosion potential and corrosion current of the coating were −0.3314 V and 22.594 × 10^−7^ A/cm^2^, respectively. With the increasing potassium titanium oxalate concentration, the corrosion potential and corrosion current decreased. When the potassium titanium oxalate concentration reached 10 g/L, the corrosion potential of the coating was −0.25743, and the corrosion current of the coating was 5.9834 × 10^−7^ A/cm^2^, lower than the potassium titanium oxalate concentration, which was 0 g/L. This indicated that the anti-corrosion property of the coating was improved.

**Figure 8:**
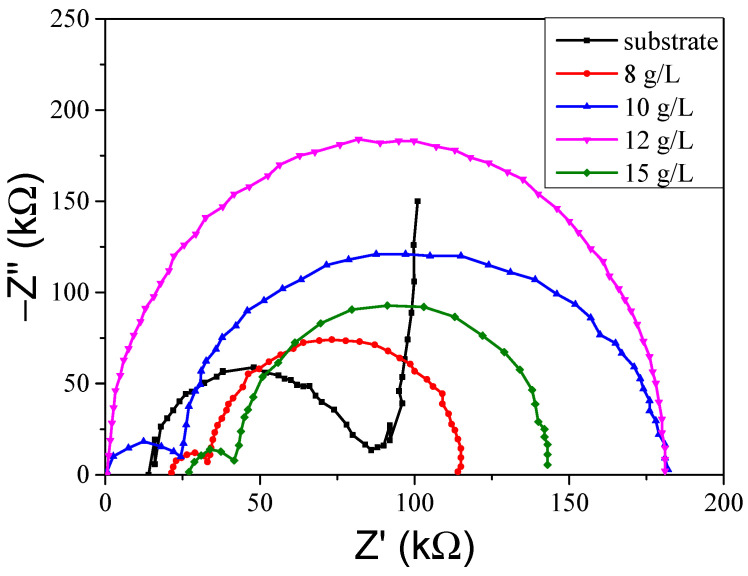
The impedance of the coatings.

To further study the influence of the potassium titanium oxalate concentration on the anti-corrosion property of the coating, the Nyquist plot of the coating was measured, and the results are shown in Figure 8. The micro arc oxide coating is composed of three layers, namely, the loose layer, the compact layer, and the transition layer [18]. Because there are many big pores in the loose layer, it is difficult to protect the substrate from corrosion [19]. It can be seen that after the micro arc oxidation treatment, the radius of the electrochemical AC impedance value was much higher than that of the aluminium alloy substrate, and an arc resistance feature was observed in the high-frequency impedance part. Because the arc radius is related to the impedance of the samples, a bigger arc radius indicated a better impedance of the sample [20]. The arc radius of the coating is bigger than the substrate, proving that the impedance of the coating is better than the substrate.

### 2.5. Influence of Titanium Oxalate Potassium Concentration on the Impedance of the Coating

**Figure 9:**
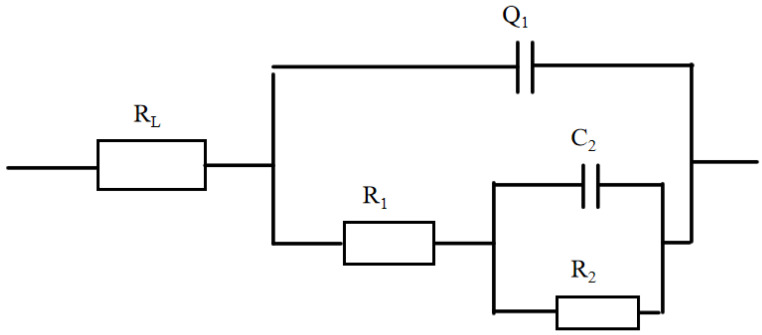
The equivalent circuit of the micro arc coatings.

To analyze the impedance on the anti-corrosion of the coating, the equivalent circuit of the coating was studied and is shown in Figure 9.

It can be observed that the impedance of the coating was composed of R_L_, Q_1_, R_1_, C_2_, and R_2_ [21], where R_L_ represented the electrolyte resistance, Q_1_ referred to the electric double-layer resistance, R_1_ denoted the loose layer resistance, C_2_ signified the substrate resistance, and R_2_ was the compact layer resistance. The results are shown in Table 3. In the table, the resistance of R_L_, Q_1_, and C_2_ was much lower than that of R_1_ and R_2_, mostly because coating impedance is studied in a 3.5% NaCl solution, and the aluminium alloy is metal. In addition, the electrolyte resistance, the substrate resistance, and electric double-layer resistance were low, whereas the loose layer resistance and compact layer resistance were high. Thus, it was found that increasing the compact layer resistance and loose layer resistance had a positive effect on improving the coating impedance [22]. The resistance of R_2_ was higher than that of R_1_, reflecting that increasing the resistance of the compact layer can clearly improve the impedance of the coating. In other words, when the potassium titanium oxalate concentration reached 10 g/L, the resistance of R_1_ and R_2_ was 18,920 Ω and 36,430 Ω, and the coating possessed the highest impedance. Therefore, the micro arc oxidation coating could protect the substrate in a better way.

## 3. Experiment

### 3.1. Materials and Reagents

Each of the experimental reagents was provided by Tianjin Kemiou Chemical Reagent Co. Ltd. (Tianjin, China). Commercial aluminium alloy 1060 (20 × 20 × 1 mm^3^) was used as a specimen.

### 3.2. Experimental Process

Two 300 × 300 mm^2^ AISI 321 stainless steel sheets were used as the cathode. The MAO process was conducted in a stirred electrolyte, consisting of 15 g/L Na_2_SiO_3_, 5 g/L KOH, and 5 g/L (NaPO_3_)_6_. Except the adjusted titanium potassium oxalate concentration of (0, 2, 5, 8, 10, 12, and 15) g/L, based on the results of previous research [23], the MAO process of the specimens was conducted at 20 °C for 30 min in a DC pulse supply with a frequency of 50 kHz, constant current density of 1 A/cm^2^, and duty cycle of 15%. A circulating water tank had been used to maintain a constant electrolyte temperature.

### 3.3. Characterization

The X-ray diffractometer (XRD; D/max-rB, RICOH, Tokyo, Japan) with CuKa sources was employed to examine the phase compositions for diverse coatings. The current applied and accelerating voltages were 30 mA and 40 kV, separately. In addition, scanning electron microscopy (SEM, S-4700, Hitachi, Tokyo, Japan) was used to investigate the cross-section and surface microstructures of the coating. Meanwhile, the equipped energy dispersive spectrometer was selected for quantitative and qualitative analysis of element points, lines, and surfaces. The open circuit voltage, Tafel curve, and electrochemical impedance of the coating were measured and analyzed by the electrochemical workstation (*Parstat* 4000, Princeton Applied Research, Oak Ridge, TN, USA). Furthermore, three-electrode systems were used, with the working electrode being the coating. The counter electrode was a platinum sheet, while saturated calomel served as the reference electrode. The contact area between the sample and the solution was 1 cm^2^, and 3.5 wt% sodium chloride was taken as a solution. The starting and ending frequencies were 100 kHz and 0.01 Hz, respectively. The samples were tested at a scanning speed of 0.2 V/s with a voltage of −2.5 V as the initial potential and ended at a voltage of 2.5 V. After testing, relevant information on the coating was obtained by fitting the Zsimp Win software, ZSimpWin 3.30.

## 4. Conclusions

(1) To improve the anti-corrosion properties of the aluminium alloy, 10 g/L titanium potassium oxalate was mixed into the electrolyte, and a micro arc oxidation coating was prepared on the surface of the aluminium alloy. The SEM and EDS results demonstrated that the compactness of the coating was improved.
(2) The results of XRD indicated that the coating consisted of Al_2_O_3_ and TiO_2_. Moreover, the electrochemical workstation was used to test the anti-corrosion of the coating, showing that the open circuit potential, corrosion potential, corrosion current, and impedance of the coating were −0.32 V, −0.32 V, 5.98 × 10^−7^ A/cm^2^, and 55 kΩ, respectively. Compared with the substrate, the corrosion potential shifted to 0.2141 V, the corrosion current decreased to 4.6246 × 10^−7^ A/cm^2^, the impedance of the coating increased to 53 kΩ, and the anti-corrosion property of the aluminium alloy was improved.

## Figures and Tables

**Table 1:** Element content (wt%) of the coating.

C_4_H_2_K_2_O_10_Ti Concentration (g/L)	Element Content				
	O	Al	Si	P	Ti
0	40.08	48.37	2.66	8.89	0
2	37.82	47.02	3.23	8.08	3.82
5	37.90	41.81	3.34	7.92	9.03
8	38.26	35.35	3.92	8.35	12.47
10	40.74	32.83	1.95	9.05	15.43
12	40.56	32.21	2.12	9.12	17.11
15	40.09	29.59	1.18	8.55	20.59

**Table 2:** The corrosion potential and corrosion current at different potassium titanium oxalate concentrations.

C_4_H_2_K_2_O_10_Ti Concentration (g/L)	Corrosion Potential (V)	Corrosion Current (×10^−7^ A/cm^2^)
substrate	−0.6215	10.608
0	−0.4754	12.594
5	−0.5139	7.8901
8	−0.4322	14.261
10	−0.4074	5.9834
12	−0.4725	9.2599
15	−0.4238	7.5732

**Table 3:** Fit values of the equivalent circuit components.

Potassium Titanium Oxalate Concentration (g/L)	Resistance (Ω)				
	R_L_	R_1_	Q_1_	C_2_	R_2_
0	0.214 × 10^−7^	91	4.659 × 10^−7^	0.6478	2174
5	65.25 × 10^−7^	154.5	1.169 × 10^−5^	0.8	280.4
8	2967 × 10^−7^	5410	3.927 × 10^−7^	0.937	154.5
10	1866 × 10^−7^	18,920	2.768 × 10^−7^	1	36,430
12	3.128 × 10^−7^	8392	1.968 × 10^−5^	0.8864	254.1
15	3.128 × 10^−7^	346.9	2.56 × 10^−5^	0.8949	1418

## Data Availability

The data presented in this study are available on request from the corresponding author.
